# Diseases of the Skin Associated with Disorders of the Female Sexual Organs

**Published:** 1889-02

**Authors:** George H. Rohé

**Affiliations:** Baltimore, Md., Professor of Hygiene and Clinical Dermatology in the College of Physicians and Surgeons, etc., etc.; No. 611 North Calvert Street


### *DISEASES OF THE SKIN ASSOCIATED WITH DISORDERS OF THE FEMALE SEXUAL ORGANS.*[1]

By GEORGE H. ROHÉ, M. D., Baltimore, Md.,

Professor of Hygiene and Clinical Dermatology in the College of Physicians and Surgeons, etc., etc.

Although disorders of the sexual apparatus, in both male and female, are not rarely considered to hold a causative relation to cutaneous eruptions, the special literature upon this subject is very indefinite and scanty. Text-books on dermatology vaguely mention uterine disturbances as etiological factors of certain skin diseases, as chloasma, urticaria and acne; while systematic works on tocology and gynecology, so far as known to the writer, are almost entirely silent upon these relations.

The writer makes no pretence to completeness in the following pages, but he has endeavored to bring together briefly many of the scattered observations on record, which seem to show a relationship or coincidence between sexual derangements and cutaneous eruptions.

The various cutaneous lesions occurring in connection with sexual disorders in females may be divided into the following classes:

1. *Angioneuroses.*
   - *a.* Edema.
   - *b.* Erythema.
   - *c.* Urticaria.
   - *d.* Acne rosacea.
2. *Disorders of the Glands.*
   - *a.* Hyperidrosis.
   - *b.* Chromidrosis.
   - *c.* Bromidrosis.
   - *d.* Seborrhea.
   - *e.* Acne simplex.
3. *Inflammations.*
   - *a.* Eczema.
   - *b.* Herpes.
   - *c.* Dermatitis herpetiformis.
   - *d.* Pemphigus.
   - *e.* Erysipelas.
   - *f.* Furunculosis.
4. *Pigmentary Hypertrophies.*
   - Chloasma.
5. *Neuroses.*
   - *a.* Pruritus.
   - *b.* Dermatalgia.
6. *Vascular Dystrophies.*
   - Purpura.

---

1. Read at the annual meeting of the American Association of Obstetricians and Gynecologists, Washington, September 20, 1888.

### I. ANGIONEUROSES.

In a recent paper, Prof. Ernst Börner, of Graz,[1] treats at some length of neurotic tumefactions (edemas) occurring as accompanying phenomena of menstruation, and the climacteric period. A considerable number of cases is reported in which evanescent or more persistent swellings of the lips, cheeks, eyelids, feet, hands, and other portions of the body, occurred contemporaneously with the menstrual period, or gave trouble during the disturbances of function classed under the designation "the change of life." In many of these cases, there were also present other neurotic manifestations, such as headache, neuralgia, or various other paresthesias. These tumefactions are similar to those reported by Quincke,[2] Jamieson,[3] and Riehl,[4] and are always related to disturbances in the sexual apparatus.

Dr. Deligny[5] gives a review of the various affections of the skin encountered in females at the time of puberty and the menopause, and enumerates hyperidrosis, erythemas, eczema, acne rosacea, pemphigus, pruritus, urticaria, pigmentary hypertrophies and erysipelas. A case of acute circumscribed edema in a neurotic dressmaker of twenty-eight years of age, is reported by Dr. E. B. Bronson,[6] but no mention is made of the condition of the genital apparatus in the patient.

Erythema multiforme, erythema nodosum, and urticaria, especially the more chronic or recurrent forms, are not infrequent accompaniments of sexual disturbances in women, occurring not only in connection with menstruation, but also as complications of diseases of the uterus.

In a case recently under the care of the writer, the patient, a neurotic girl of eighteen, suffered from an intense outbreak of urticaria at each menstrual period, although milder attacks also occurred in the intervals.

---

1. Ueber Nervöse Hautschwellungen als Begleiterscheinungen der Menstruation und des Klimax. *Volkmann's Sammlung Klinischer Vorträge, No. 312.*
2. *Monatshefte f. prakt. Dermatologie*, Bd. i., Heft 5.
3. *Edinburgh Medical Journal*, June, 1883.
4. *Wien. Med. Wochenschrift*, No. 24, 26, 1887.
5. *Le Concours Médical*, April 14, 1888.
6. *Journ. Cutan. and Gen. Urin. Dis.*, August, 1888.

Mr. Lawson Tait has recently reported a number of cases in which urticaria came on after abdominal section. Hebra first called attention to the frequent association of urticaria with uterine disorders. Scanzoni has likewise pointed out this coincidence.

The frequently recurring flushings of the face are well known to all practitioners as characteristic symptoms of the change ot life.

Acne rosacea, while it sometimes occurs at puberty, is much more frequent at the menopause. When it exists before the change of life, it is generally very much aggravated at that period, while its beginning can often be traced to the frequent congestion of the face (flushes), so constant a symptom of the climacteric.

### 2. DISORDERS OF THE GLANDS.

Dr. Deligny mentions hyperidrosis as frequent at the menopause, and more rarely at puberty. The sweating may be localized or general. Every gynecologist must have noticed the strongly odorous perspiration which is so often an accompaniment of menstrual irregularity. Colored sweat is also sometimes found in company with uterine or ovarian disorder. In this category probably belong the cases of bloody sweat, or ephidrosis cruenta, so far as the reports may be considered as trustworthy. So competent an observer as Dr. McCall Anderson has reported[1] an interesting case of this affection, and has quoted a number of others from Erasmus Wilson, T. K. Chambers, Pinel, and other authors. It is noticeable that in all but one of the cases mentioned, the points of appearance of the bloody fluid were on the front of the body, or on such portions ot the surface as could be readily reached with the hands.

Duhring[2] states that colored sweat is "not infrequently connected with uterine disorders," and gives references to a large number of reported cases.

1. *Journal Cut. Med.*, vol. i., p. 328; and Dis. of the Skin. p. 481, 1887.
2. Diseases of the Skin, 3d Ed., p. 143, 1882.

A case is described by Dr. Parvin,[1] in which there was an emergence of blood, with purplish discoloration of the lips at the menstrual periods. Other cases are referred to by Dr. Parvin, in which the mammary gland, the external auditory meatus, and the umbilicus, were the source of the bleeding. These cases are usually called vicarious menstruation, or, as Dr. Parvin proposes, xenomenia; but the question of vicarious menstruation seems to the writer still an open one. In all cases similar to those here referred to, the honesty of the patient is open to grave suspicion.

Seborrhea is often associated with menstrual derangements in chlorotic girls, at and after the period of puberty.

Acne is so frequently associated with menstruation, that every practitioner is familiar with the relationship between this skin disease and the uterine and ovarian functions. The writer thinks he has observed one form of acne which may be classified, and which he has ventured to term "menstrual acne." It differs from the ordinary form of acne in being rarely distinctly pustular, the eruption coming out in the course of a few days preceding or at the beginning of the menstrual period, and frequently disappearing with the menstrual discharge, without proceeding to suppuration. In passing, it may be mentioned that a very efficient remedy for this form of acne is arsenic in doses of one one-hundredth grain, thrice daily, combined with the external use of a two per cent. lotion of resublimed resorcine in diluted alcohol.

Acne vulgaris is usually aggravated during the menstrual period, but the eruption of new lesions does not cease in the interval, and pustulation is often very marked. In these cases, arsenic will usually be found to aggravate the eruption.

### 3. INFLAMMATIONS.

Hebra[2] speaks of the frequent coincidence between eczema and menstrual anomalies. These menstrual eczemas, he says, are especially localized on the scalp, face, or lips. In the course

1. Gynecol. Trans., vol. i., p. 135.

2. *Hautkrankheiten*, Bd. i., p. 456.

of pregnancy also, eczemas frequently appear, generally in the earlier months, and continue, in spite of all treatment, to the end of gestation. These eczemas are usually localized upon the hands. Some women, who have repeatedly been pregnant, are able to determine the presence of pregnancy in themselves by the outbreak of the eczematous eruption on their hands. Th. Veiel[1] reports an interesting case, in which the eczema appeared in the third month of the patient's third pregnancy and continued until the termination of the puerperal period, when it disappeared without treatment. The eruption was limited to the extensor surfaces of the forearms, and recurred at five consecutive subsequent pregnancies.

Hebra also refers to the eczema occurring during or after lactation. Sterile women, too, are subject to recurrent eczemas, although these may generally be traced to some defined lesion of the uterus or ovaries. The writer has noticed a form of acute, generalized, eczematous eruption not described by other writers, which occurs in association with laceration of the cervix uteri. The eruption extends over nearly the entire surface, is finely vesicular, and accompanied by the most intense itching, fever, and subsequent exfoliation of the epidermis. No treatment, addressed to the cutaneous disease, seems to be of any avail until the uterine lesion is remedied.

Climacteric eczema is referred to by Hebra, and Mr. W. Allen Jamieson, in his recent work,[2] devotes some attention to this form. He says: "Usually the monthly loss has ceased when the eczema appears. This form exhibits a proneness to relapse, and to the recurrence of eczema in certain definite regions for many years. More than three-fourths of the cases occur on the scalp and ears. The extremities also may suffer, but the trunk is scarcely affected in any case. The scaly and weeping varieties predominate, in contrast to the pustular form, which attacks infants. Itching is well marked. From the commencement to the close there may be no more than a dry pityriasis

1. *Ziemssen's Hdb. f. Spec. Path. u. Ther.*, xiv., 1, p. 304.

2. Diseases of the Skin: Edinburgh, 1888, p. 257.

eczema, with some loss of hair, liable, however, to be transformed into the moist form by external or internal irritants. Arsenic exerts considerable influence in restraining the disease, and the best local remedy seems to be the ointment of the ammoniated mercury. In some cases, however, a lotion of liquor carbonis detergens and liquor plumbi subacetatis is better."[1]

Eczema of the mammæ may also be referred to here. It is usually associated with lactation, which furnishes the irritant cause for its peculiar localization. Especial attention is claimed, however, by a peculiar form of eczematous inflammation of the mammary gland, which is especially liable to eventuate in malignant disease. This was first mentioned by Sir James Paget, and is usually described under the the name of "Paget's disease of the nipple." This affection is so important in its possible results, and so little attention has apparently been paid to it by gynecologists in this country, that no apology is necessary for quoting at some length from the original description given by Sir James Paget. This writer had seen about fifteen cases of the disease before 1874. His account of the affection is as follows:

"The patients were all women, varying in age from forty to sixty or more years, having in common nothing remarkable but their disease. In all of them the disease began as an eruption on the nipple and areola. In the majority it had the appearance of a florid, intensely red, raw surface, very finely granular, as if nearly the whole thickness of the epidermis was removed; like the surface of very acute diffuse eczema, or like that of an acute balanitis. From such a surface, on the whole or greater part of the nipple and areola, there was always copious, clear, yellowish, viscid exudation. The sensations were commonly tickling, itching, and burning, but the malady was never attended by disturbance of the general health.

"I am not aware that in any of the cases which I have seen, the eruption was different from what may be described as long-persistent eczema, or psoriasis, or by some other name, in treatises on diseases of the skin; and I believe that such cases sometimes occur on the breast, and after many months' duration are cured, or pass by, and are not followed by any other disease. But it has happened that in

---

1. ℞—Liquor plumbi subacetatis . . . ℥ss.

Liquor carbonis detergens . . . ℥ijss. Misce.

Sig.—One teaspoonful, mixed with a pint of warm water, to be applied with a sponge twice a day.

every case which I have been able to watch, cancer of the mammary gland has followed within, at the most, two years, and usually within one year. The eruption has resisted all treatment, both local and general, that has been used, and has continued even after the affected part of the skin has been involved in the cancerous disease.

"In practice, the question must be sometimes raised whether a part through whose disease or degeneracy cancer is very likely to be induced, should not be removed. In the member of a family in which cancer has frequently occurred, and who is at or beyond middle age, the risk is certainly very great, that such an eruption on the areola, as I have described, will be followed within a year or two by cancer of the breast. Should not, then, the whole diseased portion of the skin be destroyed or removed as soon as it appears incurable by milder means?" [1]

Microscopic examinations of the tissues in Paget's disease which have been made by Dr. George Thin, in England, and the late Dr. Henry Wile, in this country, seem to show that the disease is epitheliomatous at a very early stage, if not from the beginning. A number of cases which have been carefully noted by competent observers (Paget, Bulkley), show that the diagnosis between true eczema of the mammæ, and Paget's disease, is not always easy. But the fact still remains that in an individual predisposed to cancer, any persistent irritation may determine the points where the disease will localize itself. In the opinion of the writer, cancer of the breast, or of any other part, may result as the direct consequences of the irritation of a prolonged eczema. It is especially advisable, therefore, that all mammary eczemas should not be neglected, but should be cured as quickly as possible.

Prof. McCall Anderson gives a table of diagnostic points between Paget's disease and eczema of the nipple, which may aid in the differentiation.

| *Paget's Disease.* | *Eczema of the Mamma.* |
|---|---|
| 1. Occurs in women over forty years of age. | 1. Generally in women before the age of forty; especially during lactation. |
| 2. Surface affected, in typical cases, of brilliant red color, raw and granular-looking after removal of crusts. | 2. Surface not so red and raw looking, and not granular, but often punctated. |

1. St. Bartholomew's Hospital Reports for 1874.

| | |
|---|---|
| 3. When grasped between the thumb and forefinger, superficial induration often felt, "as if a penny were laid on a soft, elastic surface, and grasped through a piece of cloth." | 3. Infiltrated, but no induration. |
| 4. Edge of eruption abrupt and sharply cut, and often elevated. | 4. Edge not abrupt. Never elevated. |
| 5. Very obstinate; and only yields to extirpation or other treatment applicable to epithelioma generally. | 5. Obstinate sometimes, but yields to treatment appropriate to eczema. |

Recurrent herpes of the genitals is probably sometimes due to intra-pelvic lesions involving the cutaneous nerve supply.

The miliary eruption occurring in the course of puerperal fever, must be considered (with the so-called puerperal scarlatina) merely as a manifestation of septicemia.

The remarkable disease first described by Hebra as impetigo herpetiformis, occurs almost always associated with pregnancy or the puerperal state. It is not yet clearly established what the etiological relations of this curious affection are. A summary of a recent paper of Kaposi on the subject,[1] indicates that the views of this excellent observer are somewhat indefinite. He regards impetigo herpetiformis as "an expression of some reflex nervous and vaso-motor disturbance, and as related in this sense to chloasma, urticaria, and pemphigus (herpes) gestationis of pregnant, puerperal, and uterine cases." Dermatologists on both sides of the Atlantic are now earnestly investigating this formidable disease, and it is hoped that we may soon be able to assign it to a definite place in our classifications.

Pruriginous bullous eruptions, recurring at successive, though not necessarily consecutive pregnancies, and termed variously herpes gestationis, hydra gestationis, and pemphigus hystericus, are especially interesting in this connection. It is not yet definitely established whether the generalization of Prof. Duhring, that all the bullous lesions classed by him under the heading "dermatitis herpetiformis," is true or not. Most dermatologists are

1. Annual of Universal Medical Sciences, 1888, vol. ii.

inclined to differ from Duhring's conclusions. The latest contribution to the subject is an exhaustive paper by Dr. Brocq, of Paris, who analyzes twenty-two cases of pemphigoid eruptions occurring during pregnancy, or during the puerperal period. The striking symptoms are: General pruritus or burning sensations, attended by fever; following this, erythematous patches, especially on the extremities, and papular, vesicular, or bullous lesions, sometimes becoming purulent. In some cases there is great prostration. The appetite is not affected. The disease is not fatal, and, while not amenable to remedies during the continuance of gestation, usually disappears without treatment after the pregnancy is terminated. It presents marked contrasts to the impetigo herpetiformis of Hebra. In Dr. Brocq's very excellent monograph,[1] which came to hand after this paper had been sent to the printer, "dermatitis herpetiformis" is divided into three classes, one of which is constituted by the herpes gestationis, or pemphigus hystericus. Dr. Brocq names this "dermatite polymorphe prurigineuse recidivante de la grossesse."

Erysipelas is reported by a number of authors as occurring in greater frequency at the menopause, but it is probable that this is merely a coincidence. Some of the cases reported as erysipelas and erysipeloid are doubtless to be classed with the tumefactions and erythemata above referred to

Furunculosis is not infrequent at the menopause, and appears sometimes to have some relation to menstrual irregularities. When occurring at or after the climacteric, careful examination will often detect the presence of glycosuria, which is a rather common affection at that period of life. In young girls, furunculosis appears to be at times associated with menstrual derangements dependent upon anemia. If we consider furunculosis as an infective inflammation and necrosis, as modern pathology teaches, the relationship between disturbances of the menstrual function and furuncular eruptions is somewhat obscure. It is not improbable that the temporary lowering of vitality during menstruation may promote the infective process.

1. De la Dematite herpétiforme de Duhring, Paris, 1888.

### 4. PIGMENTARY HYPERTROPHIES.

Localized increase of the cutaneous pigment is one of the most frequent accompaniments of derangements of the generative apparatus in the female. The surface usually affected is the face, although in some cases reported the entire body has shown a marked discoloration. The commonest forms of the pigmentation are those known under the terms moth patches, mask, liver spots, chloasma uterinum, etc. They are yellowish-brown to dark brown in color, and are most frequently found on the forehead, cheeks, chin, and eyelids. They are found associated with the most varied uterine affections, and are likewise not rare in pregnancy. Women subject to menstrual irregularities are especially prone to this pigmentary hypertrophy. A number of curious cases of deep pigmentation of various parts of the body are recorded in the literature. Thus Le Cat[1] refers to a case in which the left leg became black during each pregnancy. Erasmus Wilson[2] reports a case of extensive pigmentary deposit in the skin of a woman of nineteen. The pigmentation had followed a nervous shock accompanied by menstrual irregularities and hysterical seizures. The same author[3] relates two other cases, one coincident with pregnancy, and the other with menstrual derangement following typhus fever. A singular case is quoted from Laycock:[4] "A woman was condemned to death by a Parisian mob. The 'lantern' was let down before her at the moment she was menstruating; menstruation immediately ceased. Her execution was deferred, and a few days after her skin became as black as that of a moderately black negro. The tint was deeper on the neck and shoulders than on the face; on the face and chest the tint was the same; it was less deep on the abdomen and the legs. The joints of the fingers were blacker than other parts; the soles, palms, and folds of the skin in the inguinal region paler. She became languishing (anemic). She died in 1819, aged seventy-five years, more than thirty-five years after the shock, the skin remaining dark until death."

1. Quoted by Dr. Robert Barnes: Gynecol. Trans., vol. i.
2. *Journal Cutan. Med.*, vol. iii., p 305.
3. Dis. of Skin, 5th ed., Phil., 1863.
4. See Barnes, loc. cit.

A pigmentary deposit about the eyelids is not frequent during menstruation and pregnancy. This seems to be a true pigmentation in the skin, and is not of the character called stearrhea nigricans in the books. The latter affection is probably always feigned, the pigmentation being produced by the patient herself—ink, burnt cork, lampblack, or other pigment being used. The writer has knowledge of a case of this sort in a married woman suffering from uterine catarrh accompanied by hysterical symptoms, in which the discoloration was produced by ink. The cheat was easily detected by applying a solution of oxalic acid, which startled the patient into a confession. Another case, doubtless of the same character, was recently brought to the notice of the writer. A suggestion that the malady was feigned proved an effectual bar to an examination, the patient dismissing her attending and consulting physicians, and refusing to allow a close inspection of the pigmentary deposit. Dr. Barnes[1] quotes a case from Mr. Yonge, which is unquestionably of the same character. The patient was a hysterical girl of sixteen, who had never menstruated.

The facial pigmentation of pregnancy usually disappears after the puerperium, but when it is persistent it can be readily removed (temporarily at all events) by several harmless external applications. The one most frequently successful is a combination of ammoniated mercury and subnitrate of bismuth.[2] This is applied to the affected surface in a thin layer every night, and washed off in the morning. Should it render the skin red or slightly scaly, or cause smarting or other subjective symptoms of irritation, its use may be intermitted for a few days, or the strength somewhat diminished. The epidermis is gradually exfoliated, removing the excess of pigment, and the new skin has a healthy, pinkish tint. After this result is attained, the remedy should be continued in a weaker preparation or at longer intervals. The same result may also be obtained by a solution

1. Loc. cit., from Philos. Trans., 1709.

2. ℞.—Hydrargyri ammoniat, Bismuthi subnitratis, } . . . . . . aa ʒj.

Ungt. aquæ rosæ, . . . . . . . . ℥j.—M.

Apply at night.

of mercuric bichloride (1-2 parts in 1,000), or by a one to three per cent. solution of salicylic acid.

Mention may here be made also of jaundice, which sometimes occurs as a complication of menstruation or pregnancy. This may occur in an epidemic form, as in an instance related by Dr. St. Vel, of Martinique, (quoted by various authors,) in which thirty pregnant women were attacked, of whom twenty miscarried and died. Of the children, all but one died; neither the fetuses prematurely expelled, nor those afterward born at full term, of the mothers who recovered, had any sign of jaundice. In grave or fatal sporadic cases of jaundice occurring during pregnancy, the icterus is usually a symptom of acute yellow atrophy of the liver, which seems to have a special predilection for pregnant women.

### 5. NEUROSES.

Pruritus as an accompaniment of pregnancy, or of derangements of the sexual organs, is a very frequent condition. In some persons it is a regular accompaniment of the menstrual period, but this is rare. In cases of uterine or vaginal catarrh, where acid secretions bathe the external genitals, vulvar pruritus is both frequent and intense. The same localized form of itching is often present in pregnancy, sometimes beginning early in gestation and lasting until after delivery. In certain cases persistent pruritus vulvæ is due to the accumulation of sebaceous secretions under the prepuce of the clitoris. The writer recalls a case of this sort where the most potent antipruritic remedies failed to give relief, until a careful ocular examination revealed the hidden source of irritation.

Pruritus vulvæ is a frequent attendant upon the climacteric period, and is here often associated with glycosuria. Happily, the form of glycosuria, which so often occurs at this period, does not seem to be a grave affection, but appears to yield readily to remedies and sometimes even to pass away without treatment. In not a few cases the climacteric and post-climacteric pruritus is generalized, and may sometimes be due to the nutritive disturbance of the skin occurring in advanced age.

Dermatalgia and hyperesthesia of the skin are also occasionally noted as symptoms of the hysterical state, when the latter is due to ovarian or uterine derangement, as well as when the "hysteria," so-called, is a manifestation of disease of the the central nervous system.

Certain trophic changes in the skin must, in the present state of our knowledge, be referred to a hypothetical perversion of function of an assumed system of trophic nerves. To these changes belongs that remarkable condition described as morphea by most dermatologists, and classed by others with scleroderma. In the overwhelming majority of cases, morphea occurs in women during the period of sexual activity. Out of twenty-five cases observed by Sir Erasmus Wilson, twenty were in females. In commenting upon this disproportion, this distinguished dermatologist adds: "Referring to the phenomena of the female constitution, fifteen of the twenty cases might be attributed to causes taking their origin in the uterine functions, namely, nine in pregnancy, and six in menstruation.[1]

### 6. VASCULAR DYSTROPHIES.

Purpura is rarely found as a manifestation of menstrual derangement. A case has been brought to the notice of the writer by a professional friend, in which a purpuric eruption appeared on the lower extremities every month while the menstrual flow was arrested. Many of the cases of so-called "vicarious menstruation," already referred to on a previous page, might appropriately be classed as purpuric eruptions. The fact, however, that so many substitutive hemorrhagic manifestations occur on the front of the body, or on such surfaces which are readily reached by the patient herself, renders most of these cases open to grave suspicion, and require the most careful objective examination before they can be accepted.

1. Journ. Cut. Med., vol. ii., No. 6.

No. 611 North Calvert Street.

